# Computational analysis of the interactions of a novel cephalosporin derivative with β-lactamases

**DOI:** 10.1186/s12900-018-0092-5

**Published:** 2018-10-04

**Authors:** Anna Verdino, Felicia Zollo, Margherita De Rosa, Annunziata Soriente, Miguel Ángel Hernández-Martínez, Anna Marabotti

**Affiliations:** 1 Department of Chemistry and Biology “A. Zambelli”, University of Salerno, Via Giovanni Paolo II, 132, 84084 Fisciano, SA Italy; 2 University of Rennes 1, Campus de Beaulieu, 35042 Rennes CEDEX, Rennes, France

**Keywords:** Antibiotics, Cephalosporins, Beta lactamases, Docking, Antimicrobial resistance

## Abstract

**Background:**

One of the main concerns of the modern medicine is the frightening spread of antimicrobial resistance caused mainly by the misuse of antibiotics. The researchers worldwide are actively involved in the search for new classes of antibiotics, and for the modification of known molecules in order to face this threatening problem. We have applied a computational approach to predict the interactions between a new cephalosporin derivative containing an additional β-lactam ring with different substituents, and several serine β-lactamases representative of the different classes of this family of enzymes.

**Results:**

The results of the simulations, performed by using a covalent docking approach, has shown that this compound, although able to bind the selected β-lactamases, has a different predicted binding score for the two β-lactam rings, suggesting that one of them could be more resistant to the attack of these enzymes and stay available to perform its bactericidal activity.

**Conclusions:**

The detailed analysis of the complexes obtained by these simulations suggests possible hints to modulate the affinity of this compound towards these enzymes, in order to develop new derivatives with improved features to escape to degradation.

**Electronic supplementary material:**

The online version of this article (10.1186/s12900-018-0092-5) contains supplementary material, which is available to authorized users.

## Background

The problem of the antibiotic resistance is as old as the discovery of antibiotics themselves. Indeed, already in 1940, only about a decade after the discovery of penicillin and even before its introduction in clinics, two researchers found a bacterial enzyme able to inactivate it [1]. When the use of antibiotics was diffused worldwide, the presence of resistant strains of bacteria able to inactivate these drugs became more and more evident. Nevertheless, the impact of this phenomenon in clinics was probably underestimated, at least until the “golden age of antibiotics” has continued. However, since 1970s, the decline in the discovery of novel antimicrobial molecules and the irresponsible misuse/overuse of traditional antibiotics has led to the selection of multidrug resistant bacteria (the so-called “superbugs”) that are now considered as a main threat for global public health [2]. Despite many initiatives to address this issue, new resistance mechanisms are emerging and spreading globally. Therefore, on one side, there is an absolute need for coordinated action of all countries to avoid the spread of antibiotic resistance; on the other hand, there is the urgency to focus the research either on the development of new antimicrobial molecules able to escape to the mechanisms of antibiotic resistance in bacteria, or to counteract the resistance against the most commonly used antibiotics.

β-lactams are still the most widely used class of antibiotics, because of their pharmacological advantages such as potent activity combined with low toxicity, and also for their ease of delivery and low production costs [3]. The most important mechanism of resistance against this class of molecules is the activity of β-lactamases, a family of hydrolytic enzymes whose genes are mainly located on easily transferable plasmids and, as a consequence, have rapidly disseminated through the different bacteria species [4]. These enzymes catalyze the cleavage of the β-lactam ring responsible for the bactericidal activity of these antibiotics. Based on their mechanism of action, they can be divided in two principal groups: serine β-lactamases and metallo β-lactamases. This last group hosts a Zn ion in the active site, which is coordinated to metal ligating amino acids, and the cleavage of the β-lactam ring proceeds with the aid of polarized water molecules [5]. The first group evolved from the penicillin-binding proteins (PBPs), the targets of β-lactam antibiotics, and their mechanism of action proceeds via the same acylation/deacylation mechanism involving a Ser residue in the active site. Serine β-lactamases can be divided in three classes based on sequence identity. All of them share three sequence motifs common also to PBPs, but the class A enzymes are characterized by the presence of a conserved Glu residue in the so-called Ω-loop involved in the activation of the catalytic water molecule during deacylation, whereas class C enzymes are characterized by a conserved Tyr residue replacing the corresponding Ser residue of class A in motif II, and class D enzymes have a unique N-carboxylated Lys residue in motif I [3].

In order to overcome the serine β-lactamases-mediated resistance, several inhibitors have been introduced in the clinic for a long time. These compounds act as suicide inhibitors, covalently bonding the Ser residue in the active site of the β-lactamase and permanently inactivating the enzyme [6]. Many combinations of β-lactam antibiotics/β-lactamase inhibitors are currently approved for clinical use, and many others are in late stage clinical development [7]. Most of them pair a β-lactamase inhibitor with a cephalosporin derivative for their broad-spectrum activity and less susceptivity to hydrolysis compared with the penicillin counterparts [3].

**Fig. 1:**
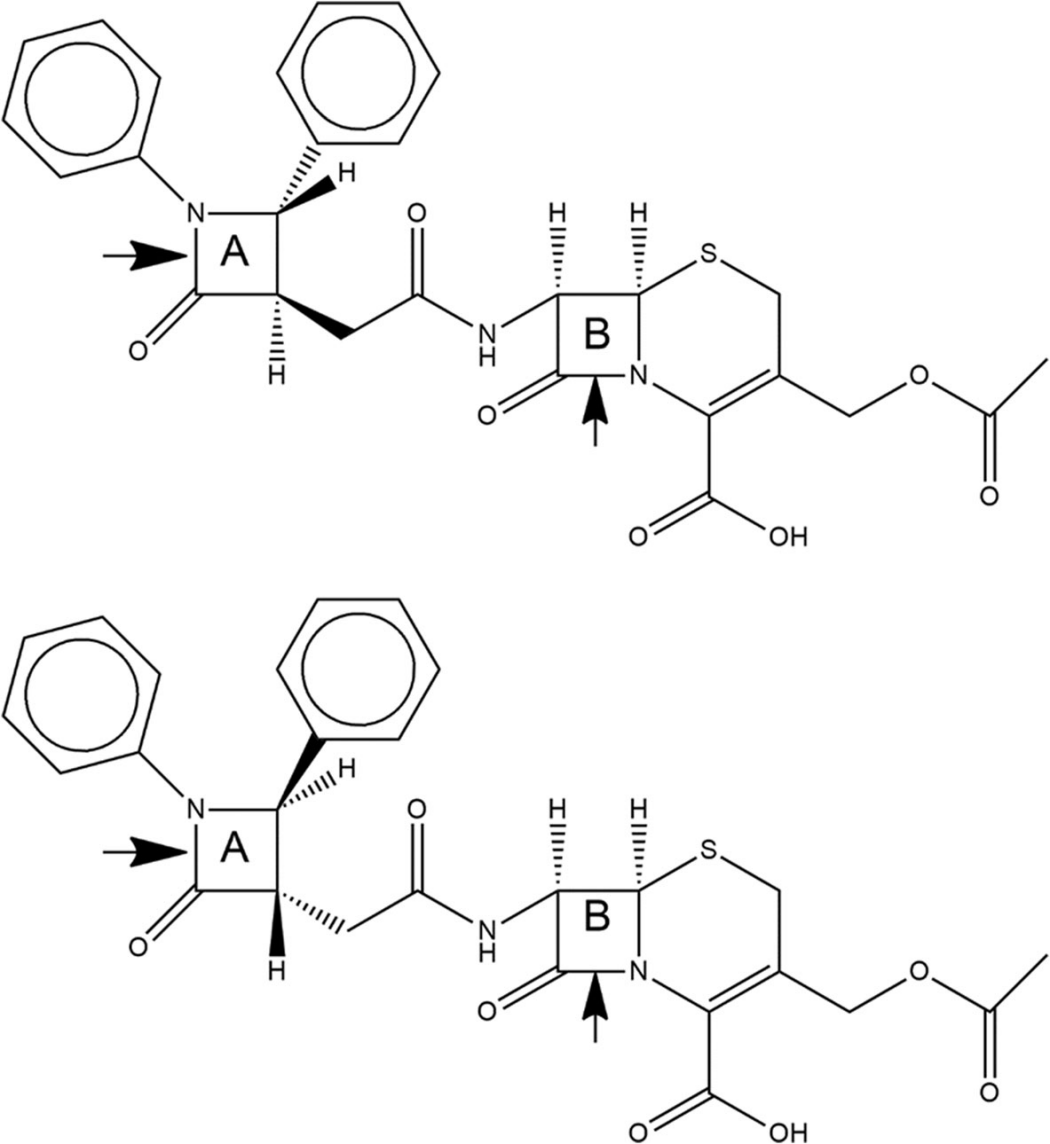
Chemical structures of the different forms of 7-ACA derivative analyzed in this study. top) Diastereoisomeric form (3R, 4S); bottom) Diastereoisomeric form (3S, 4R). A and B indicate the reactive isolated 2-azetidinone ring and the β-lactam ring of the 7-ACA nucleus, respectively. The arrows indicate the positions where the rings are opened to simulate the acylation reaction

In the last years, we have synthesized new 6-aminopenicillanic acid (6-APA) and 7-aminocephalosporanic acid (7-ACA) derivatives containing an additional β-lactam ring bound to the nitrogen of 6-APA/7-ACA scaffold via an amide bond. These compounds are active against Gram-positive bacteria at a concentration comparable with that of the reference antibiotic ceftriaxone (a third-generation cephalosporin widely used in clinics), with minimal in vitro cytotoxicity at a concentration even 10 times higher than that required for their antimicrobial effect. Moreover, the 7-ACA derivative (Fig. 1) showed better antimicrobial activity against *S. aureus*, one of the main pathogens responsible for nosocomial infections [8, 9]. An extended computational analysis of this compound allowed to study the interactions between this molecule and some candidate target PBPs. The results showed that this compound is potentially able to interact both with Gram-positive and Gram-negative enzymes, and that the 7-ACA nucleus is the preferred moiety targeting these enzymes, but also the isolated 2-azetidinone ring can interact with the active site of all PBPs. Moreover, a more favorable binding energy towards PBP2a, isolated from a multi-resistant *S. aureus* strain, was predicted for 7-ACA with respect to ceftriaxone [9]. All these data suggest that the 7-ACA derivative is a promising lead compound to develop a new class of antibiotics.

In order to collect useful information for the development of derivatives of this new compound with increased potency, wider spectrum of action and especially increased resistance to hydrolysis, we have performed an extensive computational analysis of the interactions of this 7-ACA derivative with selected β-lactamases (Table 1) [10–21]. We present here the results of these studies and we discuss possible suggestions for the rational design of future new compounds.

**Table 1:** List of the structures of serine β-lactamases selected for this study

PDB file	Ambler class^a^	Bush-Jacoby-Medeiros group^b^	Type	Organism	Features	Reference
3BLM	A	2a	PC1	*S. aureus*	Wild type	Herzberg, 1991 [10]
1XPB	A	2b	TEM-1	*E. coli*	Wild type	Fonzé et al., 1995 [11]
1ZG4	A	2b	TEM-1	*E. coli*	V84I, A184V	Stec et al., 2005 [12]
1JVJ	A	2b	TEM-1	*E. coli*	N132A	Wang et al., 2002 [13]
1NXY	A	2b	TEM-1	*E. coli*	M182 T	Wang et al., 2003 [14]
1FQG	A	2b	TEM-1	*E. coli*	E166N	Strynadka et al., 1992 [15]
3MKF	A	2b	SHV-1	*K. pneumoniae*	Wild type	Ke et al., 2011 [16]
4MBH	A	2b	SHV-1	*K. pneumoniae*	E166A	Rodkey et al., 2014 [17]
4KZ7	C	1	AmpC	*E. coli*	Wild type	Barelier et al., 2014 [18]
1L0F	C	1	AmpC	*E. coli*	N152H	Beadle and Shoichet, 2002 [19]
3LCE	D	2d	OXA-10	*P. aeruginosa*	Wild type	Johnson et al., 2010 [20]
4JF5	D	2d	OXA-23	*A. baumanii*	Wild type	Smith et al., 2013 [21]

^a^classification by Ambler et al. [22]

^b^classification by Bush et al. [23, 24]

## Results

### Interaction of 7-ACA derivative with TEM-1 β-lactamase

**Fig. 2:**
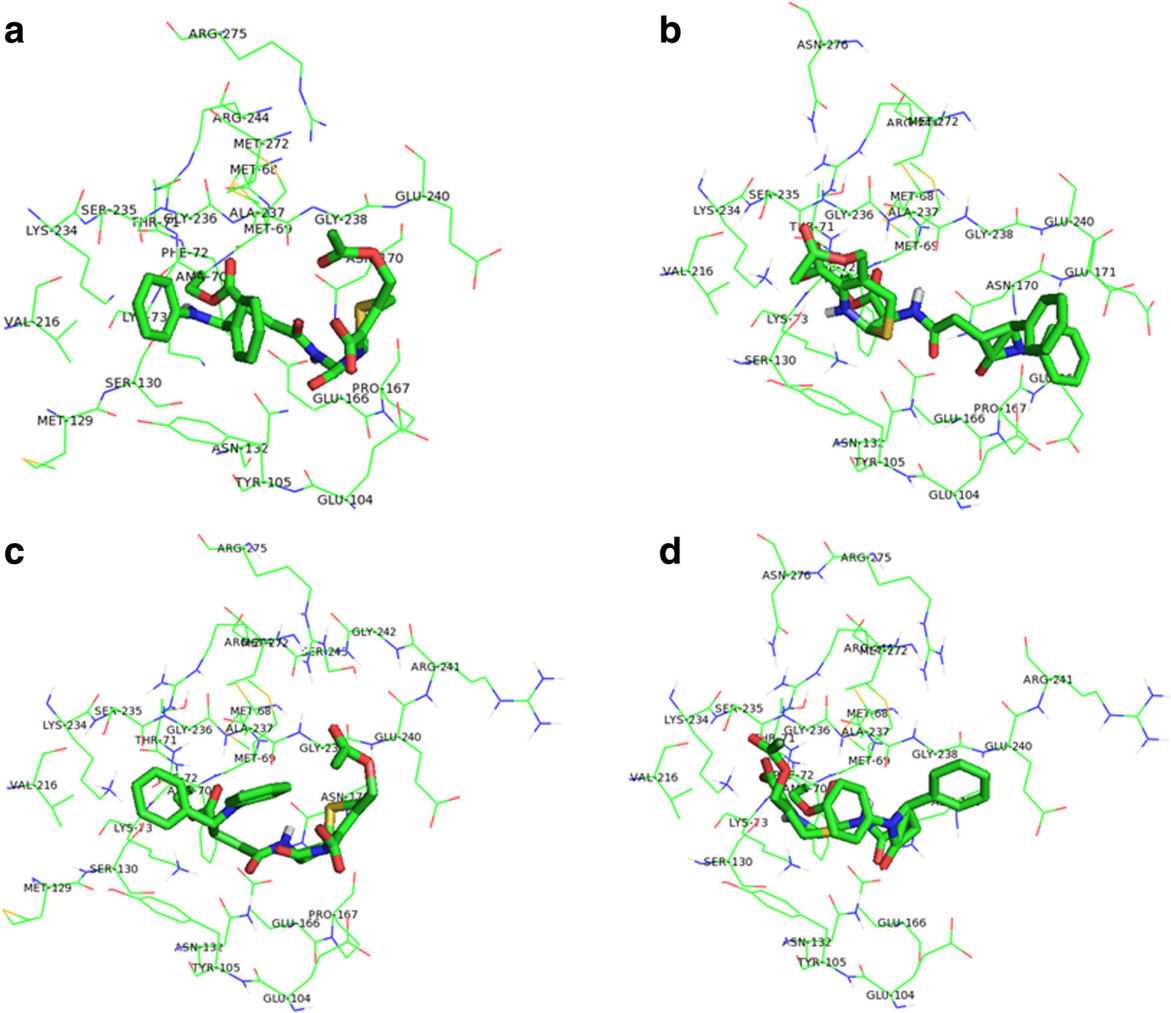
Interactions of 7-ACA derivative with the active site of wild type TEM-1 β-lactamase (PDB code: 1XPB). The poses with the lowest docking score are represented for: **a**) 7-ACA derivative (3R,4S), ring A reactive; **b**) 7-ACA derivative (3R,4S), ring B reactive; **c**) 7-ACA derivative (3S,4R), ring A reactive; **d**) 7-ACA derivative (3S,4R), ring B reactive. The 7-ACA derivative is represented in stick mode. The residues at a distance of less than 5 Å are represented as lines and labelled. The color code is: carbon green, oxygen red, nitrogen blue, sulphur yellow. Hydrogens are not shown. See Additional file 1: Table S1 for further details

The results of the simulations with this enzyme are reported in Table 2. Figure 2 shows the different diastereoisomers of the 7-ACA derivative in the binding pocket of wild type TEM-1 β-lactamase (PDB code: 1XPB). The predicted binding scores of the 7-ACA derivative for the different TEM-1 enzymes are similar to those predicted for several *E. coli* PBPs [9], and no significant differences are predicted between the wild type and mutant forms of TEM-1. The predicted binding scores of ceftriaxone, instead, tend to be lower in the case of TEM-1 with respect to PBPs [9]. Although the difference is not fully significant in absolute values, this tendency is conserved across the various PBP with respect to all TEM-1. Thus, the interaction of β-lactamases with this control drug seems to be more favored for ceftriaxone with respect to the 7-ACA derivative, suggesting that the new compound could be more resistant to β-lactamases.

**Table 2:** Results of covalent docking between 7-ACA derivative, ceftriaxone and β-lactamase TEM-1 from *E. coli*

Ligand	1XPB WT		1ZG4 V84I + A184V		1NXY M182T		1JVJ N132A		1FQG E166N	
	Predicted binding score and number of poses of the best results^a^	Total number of clusters	Predicted binding score and number of poses of the best results^a^	Total number of clusters	Predicted binding score and number of poses of the best results^a^	Total number of clusters	Predicted binding score and number of poses of the best results^a^	Total number of clusters	Predicted binding score and number of poses of the best results^a^	Total number of clusters
7-ACA derivative (3R,4S), ring A reactive^b^	Run16: − 9.78 (79)	6	Run 86: − 10.74 (86)	9	Run 32: − 10.30 (43) Run 14: − 9.99 (47)	5	Run 53: − 10.46 (89)	7	Run 66: − 10.45 (36) Run 49: − 9.27 (46)	10
7-ACA derivative (3R,4S), ring B reactive^c^	Run 93: − 13.05 (35) Run 63: − 11.92 (45)	7	Run 22: − 13.28 (53)	7	Run 91: − 12.44 (53)	8	Run 24: − 11.84 (31) Run 56: − 10.71 (42)	9	Run 59: − 13.51 (36)	9
7-ACA derivative (3S,4R), ring A reactive^b^	Run 47: − 10.39 (38)	8	Run 7: − 10.50 (59)	6	Run 91: − 10.94 (30)	6	Run 19: − 10.60 (39) Run 65: − 10.58 (51)	6	Run 67: − 9.86 (35) Run 49: − 9.68 (47)	6
7-ACA derivative (3S,4R), ring B reactive^c^	Run 61: − 12.45 (49)	9	Run 77: − 13.50 (51)	9	Run 2: − 12.23 (2) Run 39: − 11.74 (56)	8	Run 54: − 12.35 (43)	7	Run 65: − 12.56 (36)	9
Ceftriaxone	Run 26: − 12.92 (14) Run 60: − 8.27 (28)	18	Run 21: − 13.64 (28)	11	Run 46: − 13.09 (10) Run 28: − 13.06 (26)	12	Run 35: − 13.04 (16) Run 45: − 12.26 (25)	15	Run 94: − 12.95 (16) Run 4: − 12.22 (24)	18

^a^when the pose with the best predicted binding score is not coincident with the one with the most populated cluster, the energy and the number of poses of the most populated cluster is additionally reported

^b^the isolated β-lactam ring has been considered reactive towards the acylation

^c^the β-lactam ring of the 7-ACA moiety has been considered reactive towards the acylation

As already shown for the interactions with PBPs [9], the two diastereoisomeric forms of the 7-ACA derivative show no significant differences in the interaction with these enzymes, confirming that their active sites are not selective towards this property. Similarly, the predicted binding score when the isolated 2-azetidinone ring is reactive towards the acylation is consistently higher than the score predicted when the β-lactam ring belonging to the 7-ACA moiety is the target for the acylation reaction. This suggests that the isolated ring is less susceptive to the formation of the Ser-acylated intermediate also towards β-lactamases. This is interesting, considering that this would render this moiety more resistant towards the attack of β-lactamases.

**Fig. 3:**
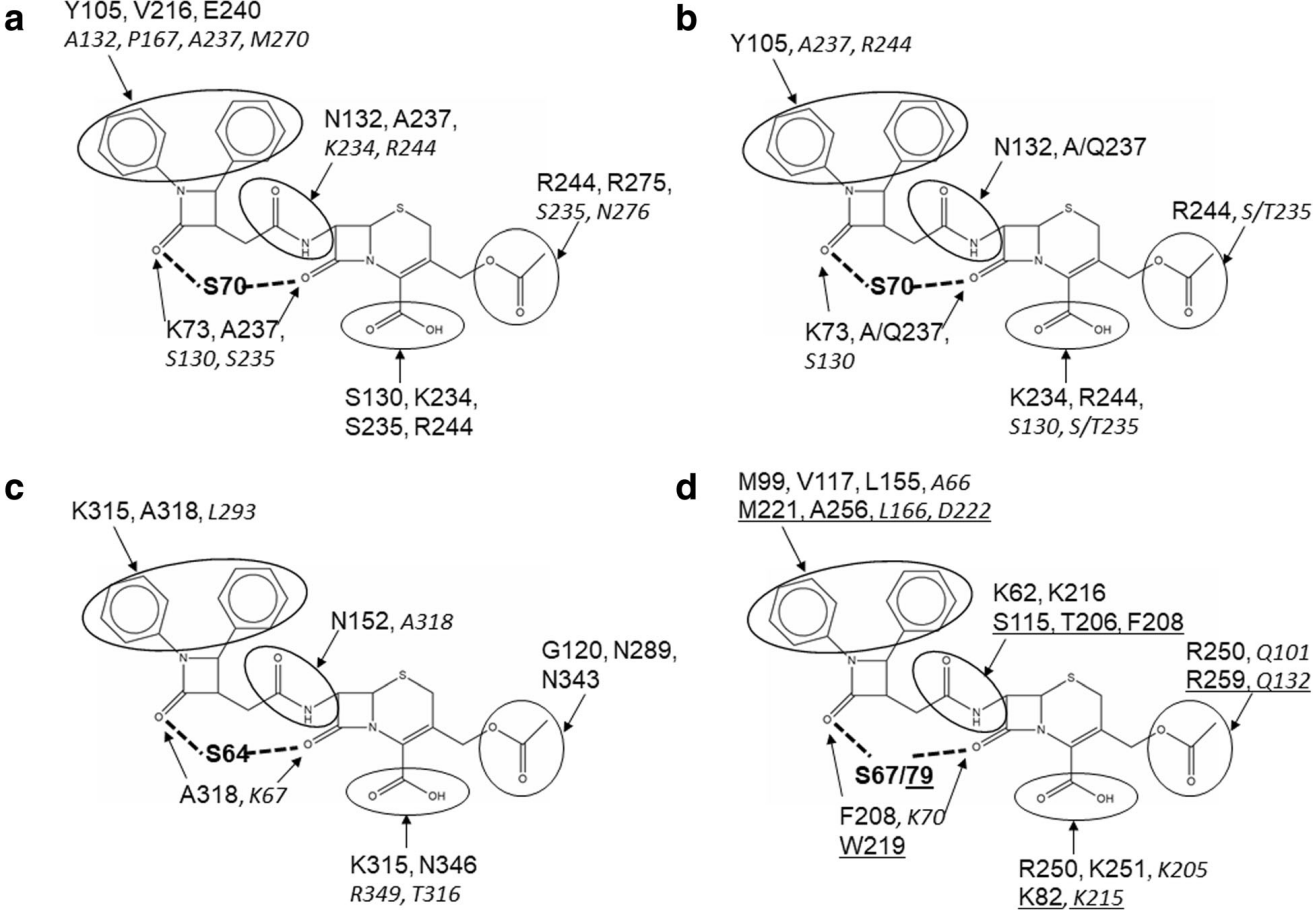
Representation of the interactions of 7-ACA derivative with the active sites of different β-lactamases. **a**) TEM-1; **b**) other class A β-lactamases; **c**) AmpC; **d**) Class D β-lactamases. The reactive Ser residue is indicated in bold, bound to one of the two possible reactive β-lactam rings (dotted line). The residues interacting more frequently with the groups discussed in the text (included in the circles) are shown in normal character, whereas less frequent interactions are shown in italics. In panel **d**, the residues belonging to OXA-10 are underlined. See text and Additional file 1: Table S1-S3 for further details

The list of the interactions between the 7-ACA derivative and the binding pocket of the different forms of TEM-1 is reported in Additional file 1: Table S1. As for PBPs, H-bonds are prevalent among other kinds of bonds, which is expected considering that many polar residues are present in the active site, interacting with the polar moieties of the antimicrobial compounds. In details (Fig. 3a), the carboxylic moiety of the 7-ACA interacts with positively charged residues (in particular, Lys234 and Arg244), but also with Ser235 and Ser130, whereas the acetoxymethyl moiety in position 3 of the 7-ACA nucleus interacts mainly with Arg244 or Arg275. The carbonyl of the acetylated Ser70 residue interacts usually with Lys73 and Ala237, and additionally with polar residues such as Ser130 and Ser235. Ala237 also interacts with the NH moiety of the amide group connecting the two halves of the 7-ACA derivative, whereas the carbonyl moiety of this group interacts frequently with Asn132. This interaction is lost in the mutant p.Asn132Ala (PDB code: 1JVJ) [13], but apparently this does not affect the predicted binding score for this protein (see Table 2). Finally, the two phenylic moieties on the isolated 2-azetidinone ring form several interactions with mainly hydrophobic residues (in particular, Tyr105) but they are also involved in interactions with charged residues.

Compared with ceftriaxone, these interactions are similar and involve the same residues, confirming that the 7-ACA derivative interacts in the same binding site of the known cephalosporin.

### Interaction of 7-ACA derivative with other selected class A β-lactamases

The results of the simulations between the 7-ACA derivative and this group of class A β-lactamases is reported in Table 3. The predicted binding scores of the interactions are in the same range with respect to those predicted for TEM-1, and no differences are present for the interaction with the enzyme belonging to the Gram-positive microorganism with respect to the enzymes from the Gram-negative microorganisms. This result is in line with our previously published results [9]. Also in this case, the most reactive moiety to acylation seems to be the β-lactam ring belonging to the 7-ACA moiety, whereas the isolated β-lactam ring appears to be less prone to acylation, considering that the predicted binding score in this case is higher, thus less favorable. The predicted binding score for ceftriaxone used as a control is similar to the one predicted for the cephalosporanic moiety of the 7-ACA derivative, and lower than that of the isolated 2-azetidinone ring. The comparison with the results previously published on PBPs from *S. aureus* [9] show that the predicted binding score of both the 7-ACA derivative and ceftriaxone for PC-1 is in line with that predicted for PBP4, but higher than the one predicted for PBP3. Therefore, in this case it seems that both antimicrobial molecules are more reactive towards their natural target proteins than being themselves a target for the β-lactamases.

**Table 3:** Results of covalent docking between 7-ACA derivative, ceftriaxone and other selected class A β-lactamases

Ligand	3BLM PC-1 from *S. aureus* WT		3MKF SHV-1 from *K. pneumoniae* WT		4MBH SHV-1 from *K. pneumoniae* E166A	
	Predicted binding score and number of poses of the best result^a^	Total number of clusters	Predicted binding score and number of poses of the best result^a^	Total number of clusters	Predicted binding score and number of poses of the best result^a^	Total number of clusters
7-ACA derivative (3R,4S), ring A reactive^b^	Run 13: − 10.33 (16) Run 60: − 9.75 (36)	10	Run 20: − 10.87 (14) Run 86: − 10.42 (42)	6	Run 17: − 10.92 (35)	13
7-ACA derivative (3R,4S), ring B reactive^c^	Run 18: − 12.44 (7) Run 37: − 12.35 (55)	11	Run 41: − 12.09 (72)	7	Run 58: − 11.80 (35)	10
7-ACA derivative (3S,4R), ring A reactive^b^	Run 100: − 10.64 (55)	10	Run 11: − 11.01 (37) Run 69: − 10.62 (33)	6	Run 13: − 9.61 (21) Run 71: − 9.39 (35)	10
7-ACA derivative (3S,4R), ring B reactive^c^	Run 23: − 12.61 (23) Run 66: − 12.60 (31)	13	Run 54: − 12.37 (25) Run 56: − 11.91 (39)	8	Run 29: − 11.78 (18) Run 5: − 11.23 (22) Run 79: − 11.02 (22)	15
Ceftriaxone	Run 94: − 12.42 (13) Run 16: − 12.33 (30)	21	Run 62: − 13.52 (21) Run 82: − 13.29 (43)	13	Run 55: − 11.84 (19) Run 38: − 11.76 (28)	16

^a^when the result with the best predicted binding score is not associated to the one with the most populated cluster, the energy and the number of poses of the most populated cluster is additionally reported

^b^the isolated β-lactam ring has been considered reactive towards the acylation

^c^the β-lactam ring of the 7-ACA moiety has been considered reactive towards the acylation

The list of the interactions in the binding pocket of these two proteins (Additional file 1: Table S2) shows that, in analogy with TEM-1, hydrogen bonds prevail for the interaction of the antibiotics. The residues of PC-1 and SHV-1 involved in the interactions with the 7-ACA derivative are structurally equivalent to those of TEM-1, and this indicates that the binding mode of this compound is similar in class A β-lactamases. Also in these cases (Fig. 3b), the carboxylic moiety of 7-ACA interacts with positively charged residues such as Lys234 and Arg244, and less frequently, with polar residues such as Ser/Thr235 and Ser130. The acetylated moiety bound to the reactive Ser70 interacts constantly with Lys73 and Ala/Gln237, and the carbonyl belonging to the amidic group in the chain connecting the two halves of the 7-ACA derivative interacts mainly with Asn132, whereas the NH moiety is constantly bound to Ala/Gln237. The phenyl groups bound to the isolated 2-azetidinone ring of the 7-ACA derivative can promote many different interactions with several hydrophobic residues such as Tyr105, Gly238, Gly236, but also with positively charged residues such as Arg244. In SHV1, residue 166 seems not to be involved in the interaction with the substrate; therefore, the results are not significantly different between the wild type and the mutant protein.

### Interaction of 7-ACA derivative with other selected β-lactamases

The results of the simulations made with AmpC β-lactamase are shown in Table 4. Despite this protein is classified as a specific cephalosporinase, the predicted binding scores are in line with those predicted for the other classes of proteins. Also in this case, the β-lactam ring belonging to the 7-ACA moiety is generally predicted to be the most reactive to acylation. However, in the mutant AmpC, the predicted difference in the binding score between the two β-lactam rings appears to be not relevant, contrarily to the wild type form. The same is true also for ceftriaxone, suggesting (in line with experimental data [19]) that the mutation is able to perturb the binding of these antibiotics to the protein.

**Table 4:** Results of covalent docking between 7-ACA derivative, ceftriaxone and other selected β-lactamases

Ligand	4KZ7 AmpC from *E. coli* WT		1L0F AmpC from *E. coli* N152H		3LCE OXA-10 from *P. aeruginosa* WT		4JF5 OXA-23 from *A. baumanii* WT	
	Predicted binding score and number of poses of the best result^a^	Total number of clusters	Predicted binding score and number of poses of the best result^a^	Total number of clusters	Predicted binding score and number of poses of the best result^a^	Total number of clusters	Predicted binding score and number of poses of the best result^a^	Total number of clusters
7-ACA derivative (3R,4S), ring A reactive^b^	Run 84: − 10.61 (29)	10	Run 79: − 11.12 (45)	9	Run 92: − 10.67 (11) Run 52: − 10.11 (28)	13	Run 95: − 10.52 (15) Run 82: − 8.62 (19)	15
7-ACA derivative (3R,4S), ring B reactive^c^	Run 1: − 14.45 (71)	7	Run 72: − 12.27 (28) Run 57: − 11.99 (28)	9	Run 17: − 12.76 (43)	13	Run 36: − 13.66 (12) Run 40: − 12.48 (28)	17
7-ACA derivative (3S,4R), ring A reactive^b^	Run 95: − 12.22 (56)	9	Run 93: − 11.06 (44)	14	Run 12: − 10.19 (15) Run 48: − 9.42 (17)	14	Run 25: − 11.77 (7) Run 95: − 9.56 (23)	14
7-ACA derivative (3S,4R), ring B reactive^c^	Run 51:− 13.48 (13) Run 42: − 11.97 (21)	13	Run 72: − 11.68 (8) Run 33: − 10.14 (26)	11	Run 99: − 11.99 (26) Run 8: − 11.55 (29)	14	Run 69: − 14.47 (26)	16
Ceftriaxone	Run 10: −12.35 (27)	19	Run 87: −11.08 (47)	16	Run 1: −12.99 (6) Run 44: −11.90 (37)	17	Run 52: −12.17 (2) Run 35: − 10.59 (17)	34

^a^when the result with the best predicted binding score is not associated to the one with the most populated cluster, the energy and the number of poses of the most populated cluster is additionally reported

^b^the isolated β-lactam ring has been considered reactive towards the acylation

^c^the β-lactam ring of the 7-ACA moiety has been considered reactive towards the acylation

The analysis of the interactions in the binding pocket is reported in Additional file 1: Table S3 and Fig. 3c. Interactions with positively charged residues such as Lys315 are still predicted for the carboxylic moiety of 7-ACA, in addition to the interaction with polar residues such as Asn346 and Thr316. The acetylated moiety bound to the reactive Ser64 residue interacts constantly with Ala318 (which contacts also the NH moiety of the amidic group of the chain connecting the two halves of the 7-ACA derivative) and, less frequently, with Lys67. Noteworthy, in wild type AmpC the carbonyl moiety of the amidic group interacts with residue Asn152, but its replacement with a His residue in the mutant enzyme does not seem to cause the loss of this interaction. Moreover, contrarily to the class A β-lactamases, the NH moiety of this amidic group does not seem to be involved in polar interactions. The phenyl groups bound to the isolated 2-azetidinone ring of the 7-ACA derivative can promote several interactions with both hydrophobic and polar residues.

For OXA-10, the predicted binding score for ceftriaxone is in line with the predicted binding score for the β-lactam ring belonging to the 7-ACA moiety, whereas in the case of OXA-23, ceftriaxone has a predicted binding score higher with respect to this moiety of the 7-ACA derivative (Table 4). Thus, in this case, ceftriaxone seems to be less susceptive to acylation and consequent deactivation of its antibacterial activity than the 7-ACA derivative. The analysis of the interactions with residues in the active site (Additional file 1: Table S3 and Fig. 3d) shows that the interactions with the carboxylic moiety of 7-ACA are similar to those of the previous enzymes, whereas those with the acetylated Ser residue and the amidic group in the chain connecting the two halves of the 7-ACA derivative are rather different. In particular, the interaction with the reactive Ser involves in both OXA enzymes a hydrophobic residue (Phe208 for OXA-10 and Trp219 for OXA-23). In OXA-10, no polar interactions are predicted for the NH moiety of the amidic group of the chain connecting the two halves of the 7-ACA derivative, similarly to the class C β-lactamase AmpC, whereas in OXA-23 this moiety contacts Phe208. The carbonyl moiety forms hydrogen bonds with residues of the binding site only in the case in which the acylation involves the isolated 2-azetidinone ring. For OXA-10, the interactions occur preferably with Lys62 and Lys216, whereas in OXA-23, Ser115 and Thr206 mediate them. Instead, the phenyl groups bound to the isolated 2-azetidinone ring of the 7-ACA derivative can promote many different interactions mainly with hydrophobic residues.

## Discussion

In our previous paper [9] we have tested this new 7-ACA derivative for its antibiotic activity and we have performed a computational study to dissect its interactions with its specific biological targets PBPs. In this study, we have extended the computational approach to serine β-lactamases in order to gain insight for the future development of antibiotics resistant to these enzymes. We have included in our study at least one representative for each Ambler class of serine β-lactamases [22], according also to the quality criteria that we have previously identified [9]. Moreover, most of the β-lactamases included in the current study are expressed by bacteria belonging to the group of the so-called “ESKAPE” pathogens, i.e. those bacteria belonging both to Gram-positive and Gram-negative species (the acronym “ESKAPE” has been created from the initial letters of *Enterococcus faecium, Staphylococcus aureus, Klebsiella pneumoniae, Acinetobacter baumanii, Pseudomonas aeruginosa, Enterobacter* species), which are leading cause of nosocomial infections throughout the world. These pathogens can be commonly found in the human microbiota and in the environment, and under the selective pressure provoked by the use (and misuse/abuse) of antibiotics, they were particularly able to accumulate multiple different antimicrobial resistance mechanisms carried by bacterial chromosomes, plasmids or genetic transposable elements. In this way, they are now able to escape the most common antimicrobial treatments and, particularly in healthcare settings due to the high concentration of infected people, they can be transmitted among individuals or they can colonize immunocompromised patients, becoming one of the principal causes of death of hospitalised patients [25].

TEM-1 was the first β-lactamase identified in *E. coli* [26]. The proteins of this family are encoded generally by plasmidic genes and are commonly detected in *E. coli* based infections. TEM-1 belongs to Ambler class A and to group 2b according to Bush-Jacoby-Medeiros classification [23, 24] and is active against both penicillins and cephalosporins. The crystal structure of wild type TEM-1 β-lactamase selected for the present work has been determined at 1.9 Å resolution in 1995 [11]. Similarly to other known β-lactamases of the same class, it consists of two domains, with the active site located at the interface between them. In addition to the wild type, in order to improve our knowledge about the binding of 7-ACA derivative with this family of enzymes, we also simulated the interaction of our cephalosporin derivative with some mutants whose 3D structure is available. In particular, we selected the structure of the double mutant p.Val84Ile + p.Ala184Val, considered as a high resolution “wild type” enzyme [12]; of TEM-1 p.Asn132Ala, a mutant that shows a dramatic change in stability when bound to inhibitors such as moxalactam and imipenem [13]; of p.Met182Thr, which is isofunctional but more stable and diffracts to a higher resolution with respect to the wild type TEM-1 [14]; finally, of p.Glu166Asn, a deacylation-defective mutant of this β-lactamase [15].

The PC-1 β-lactamase used in this study derives from the Gram positive organism *S. aureus,* belonging to the ESKAPE group [25]. It is classified by Ambler as a class A serine penicillinase and is more specific for penicillins, being classified in the group 2a according to Bush, Jacoby and Medeiros. The structure selected for the present work has been determined at 2.0 Å [10] and, similarly to TEM-1, it consists of two closely associated domains with the active cleft located between them.

SHV-1 is another β-lactamase belonging to class A and group 2b, sharing approximately 68% sequence identity with TEM-1 [27]. It can be considered another common β-lactamase, found primarily in *K. pneumoniae* (another bacterial species belonging to the ESKAPE group) [25]. We have selected two structures for our study: the wild type protein, solved at a resolution of 1.33 Å in a complex with a boronic acid transition state inhibitor [16], and that of the mutant p.Glu166Ala complexed to a penam sulfone, solved at high (1.22 Å) resolution [17]. This last mutant, similarly to the mutant p.Glu166Asn of TEM-1, is a deacylation deficient variant.

AmpC from *E. coli* has been selected as a representative enzyme for class C β-lactamases (cephalosporinases, i.e. specific for cephalosporins)*.* More than 100 structures are present in PDB for this protein, and we have selected the one of the wild type protein determined at 1.43 Å [18] and the structure of the mutant p.Asn152His, involving one of the catalytic residues, whose mutation determines the impairment of the enzymatic activity and a concomitant enhancement of the stability of the enzyme [19].

Finally, we have selected two representative structures of β-lactamases belonging to class D of Ambler classification (the so-called oxacillinases): OXA-10 from *P. aeruginosa* and OXA-23 from *A. baumanii* [20, 21]. Both organisms belong to the ESKAPE group, and in particular, the latter one, regarded as of little clinical importance just a decade ago, has been recently re-classified as a major opportunistic pathogen [25]. The rapid spread of the OXA-23-producing pathogens in the clinics worldwide constitutes a serious threat to human health [21]. The structure of OXA-10 has been determined at 2 Å in complex with a cyclobutanone analogue of β-lactams [20]. The structure of OXA-23 has been determined in complex with meropenem, a clinically important carbapenem antibiotic [21].

The interactions predicted for the 7-ACA derivative show several features in common within all these different β-lactamases and with the PBPs previously tested [9]. The predicted binding scores are in the same range of amplitude for the two families of enzymes, but the two β-lactam rings show different reactivity towards acylation, being the isolated 2-azetidinone ring the less reactive one in both cases. This would therefore increase the ability of this compound to escape from the inactivation of β-lactamases, conserving a reactive moiety for the interaction with PBPs. We are investigating if the different reactivities of these two rings could be modulated by their reciprocal distance in the molecule, by synthesizing and testing derivatives with a longer spacer. The polar interactions with the carboxylic moiety of 7-ACA and with the amide moiety in the chain connecting the two halves of this compound appear to be well conserved, although in the case of class D β-lactamases they involve hydrophobic residues. The two phenyl rings seems to be important for the interaction with different residues of the active site, and we think that the introduction of substituents could modulate the affinity of the 7-ACA derivative for the different enzymes.

## Conclusions

By using an innovative computational approach, we have performed a deep characterization of the interaction of a new cephalosporin derivative bearing an additional 2-azetidinone ring bound to the 7-ACA nucleus, with serine β-lactamases belonging to different classes, in order to collect information about the potential ability of this compound to be more resistant to their inactivation. The results show that the interaction between this class of enzymes and the isolated β-lactam ring is less favored with respect to the interaction with the “canonical” 7-ACA moiety, with no significant differences between the two diastereoisomeric forms obtained from the chemical synthesis of this compound [9]. The isolated ring seems therefore less prone to the catalytic cleavage of these enzymes, and this would improve the resistance of this class of compounds towards inactivation. The detailed analysis of the interactions between the 7-ACA derivatives and the residues of the active site of the selected β-lactamases suggests that the two phenyl rings bound to the isolated 2-azetidinone moiety and the amidic group that connects this moiety to the 7-ACA nucleus could be modified in order to modulate the affinity of this compound for these enzymes.

This study represents an important step to develop a new class of antibiotics hopefully able to overcome the bacterial resistance, thus becoming a new, effective weapon in the battle for survival against microbes that we cannot absolutely risk to loose.

## Methods

### Selected β-lactamases structures

Many structures of serine β-lactamases are available in the Protein Data Bank [28]. The selection of representative proteins for the present work was made on the basis of different quality criteria including: good resolution (< 2 Å), R-value and R-free parameters, absence of missing residues and atoms at least in the active site, analysis of distribution of residues into the Ramachandran plot and of the RMS deviation of bond lengths and angles from ideal values, low average and local B-values. Additionally, two web servers were used to evaluate the quality of the structures: ProSA-Web [29] and Q-MEAN [30]. The proteins selected for this study belong to both Gram-positive and Gram-negative organisms and represent some of the most studied β-lactamase families. For some of these proteins, we analyzed both the wild type and selected available mutant forms.

### Structure of the 7-ACA derivative and ceftriaxone

The structure of the 7-ACA derivative tested in the present work was designed and saved in 3D .pdb format by using ChemDraw and Chem3D Pro 12.0 (Perkin Elmer). Since the chemical synthesis of this compound produced a diastereoisomeric mixture [9], we designed and analyzed separately the two diastereoisomers (3R, 4S) and (3S, 4R). Moreover, since both 2-azetidinone rings can be potential target for the acylation/deacylation reaction, for each diastereoisomeric form we have designed and analyzed separately the form with the isolated β-lactam ring (called ring A) open and reactive to acylation, and the form with the β-lactam ring belonging to the 7-ACA nucleus (called ring B) open and reactive to acylation (Fig. 1). Finally, we considered the 7-ACA moiety both in dissociated and undissociated form, but since the results were not significantly different, we report here the results of the undissociated form only.

The structure of ceftriaxone, used as reference cephalosporin derivative, was downloaded from PubChem [31] in 3D .sdf format, then converted in the .pdb format by using Chimera [32]. Chimera was also used to open the β-lactam ring and modify the ligand to prepare it for covalent docking (see below) and, if necessary, to optimize the modified structure for the following steps.

### Covalent docking simulations and analysis

The simulation of the interaction of 7-ACA derivative with the selected β-lactamases was performed by applying the covalent docking approach based on flexible side chain method recently developed by Bianco and colleagues [33] implemented in the popular program AutoDock v.4.2 [34], previously applied to the study of the interactions of the 7-ACA derivative with PBPs [9].

For this procedure, the ligand must be first modified by opening the β-lactam ring and adding the C=O group at the site of acetylation. This is needed to simulate the formation of the covalent bond with the Ser residue in the active site, as these atoms are overlapped with the matching atoms in the receptor structure. The resulting complex is treated as a fully flexible residue in the protein, and the following steps mimic a traditional flexible docking procedure in which the position and conformation of the flexible residue is optimized. This method has 75% success rate in reproducing the experimental coordinates of covalent complexes in a test set created by the authors [33].

Before proceeding with the study of 7-ACA derivative and ceftriaxone, a self-docking strategy was applied to check the reliability of the procedure and to obtain an estimate of the predicted binding score in the case of known antibiotics, by simulating the docking of penicillin in the crystallographic structure of TEM-1 β-lactamase (PDB file: 1FQG) [15]. The result obtained was then compared with the original crystallographic structure (Additional file 1: Figure S1) and proved to be fully superimposed (the RMSD between the coordinates of the crystallographic structure and of the best pose is 0.072 Å), thus confirming the validity of this approach.

For the study of the interactions of 7-ACA derivative and ceftriaxone with the selected β-lactamases, polar hydrogens were added to the protein and the ligands, and charges were assigned according to Gasteiger [35]. Water molecules and other ligands present in the crystallographic structures were removed before simulations. A grid centered on the catalytic Ser residues and including those residues belonging to the active site reported for each β-lactamase was set up, with a maximum dimension of 70x70x70 points and a grid spacing of 0.375 Å. For each docking simulation, 100 runs were performed using the AutoDock Lamarckian algorithm and leaving all the other parameters as default. The conformations corresponding to the lowest docking binding score (calculated with a semiempirical free energy force field including terms for dispersion/repulsion, hydrogen bonding, electrostatics, and desolvation [36]) and the most populated cluster of poses were then selected, saved in .pdb format and the interactions between the β-lactamase and the 7-ACA derivative or ceftriaxone were investigated by using the tools available in the Discovery Studio software (DassaultSystèmes BIOVIA, Discovery Studio Modeling Environment, Release 4.5 San Diego, 2015).

## Additional file

Additional file 1: **Table S1.** List of the interactions between 7-ACA derivative, ceftriaxone and the residues in the binding pocket of the different TEM-1 β-lactamases from *E. coli*. In this table are reported for each protein the interactions of the representative pose(s) reported in Table 2. **Table S2.** List of the interactions between 7-ACA derivative, ceftriaxone and the residues in the binding pocket of the class A β-lactamases different from TEM-1. In this table are reported for each protein the interactions of the representative pose(s) reported in Table 3. **Table S3.** List of the interactions between 7-ACA derivative, ceftriaxone and the residues in the binding pocket of the class C and D β-lactamases. In this table are reported for each protein the interactions of the representative pose(s) reported in Table 4. **Figure S1.** Superposition between the representative run obtained by covalent self-docking (red) and the crystallographic structure of the complex (blue) of TEM-1 β-lactamase with penicillin (PDB file; 1FQG). The RMSD calculated on the two structures is 0.072 Å. (DOCX 97 kb)

## Abbreviations

6-APA: 6-aminopenicillanic acid
7-ACA: 7-aminocephalosporanic acid
PBPs: Penicillin binding proteins
