# Supplementary material for: Computational analysis of the interactions of a novel cephalosporin derivative with β-lactamases
Source: BMC Struct Biol. 2018 Oct 4;18:13. doi: 10.1186/s12900-018-0092-5 (PMC6389238; doi:10.1186/s12900-018-0092-5)
Supplement: Supplementary file 1 — Table S1. List of the interactions between 7-ACA derivative, ceftriaxone and the residues in the binding pocket of the different TEM-1 β-lactamases from E. coli. In this table are reported for each protein the interactions of the representative pose(s) reported in Table 2. Table S2. List of the interactions between 7-ACA derivative, ceftriaxone and the residues in the binding pocket of the class A β-lactamases different from TEM-1. In this table are reported for each protein the interactions of the representative pose(s) reported in Table 3. Table S3. List of the interactions between 7-ACA derivative, ceftriaxone and the residues in the binding pocket of the class C and D β-lactamases. In this table are reported for each protein the interactions of the representative pose(s) reported in Table 4. Figure S1. Superposition between the representative run obtained by covalent self-docking (red) and the crystallographic structure of the complex (blue) of TEM-1 β-lactamase with penicillin (PDB file; 1FQG). The RMSD calculated on the two structures is 0.072 Å. (DOCX 97 kb) [file 12900_2018_92_MOESM1_ESM.docx]

**Additional file**

**Computational Analysis of the Interactions of a Novel Cephalosporin Derivative with β- Lactamases**

Anna Verdino^1^, Felicia Zollo^1^, Margherita De Rosa^1^, Annunziata Soriente^1^, Miguel Angel Hernandez-Martinez^2^, Anna Marabotti^1,^*

^1^: Department of Chemistry and Biology "A. Zambelli", University of Salerno, Via Giovanni Paolo II, 132, 84084 Fisciano (SA), Italy

^2^: University of Rennes 1, Campus de Beaulieu, 35042 Rennes CEDEX, France

*Corresponding author. Phone: +39 089 969583. E-mail: [amarabotti@unisa.it](mailto:amarabotti@unisa.it). ORCID: 0000-0002-9510-5205

**Table S1: List of the interactions between 7-ACA derivative, ceftriaxone and the residues in the binding pocket of the different TEM-1 β-lactamases from *E. coli*.** In this table are reported for each protein the interactions of the representative pose(s) reported in Table 2.

| **Ligand** | **1XPB**  **WT** | **1ZG4**  **V84I+A184V** | **1NXY**  **M182T** | **1JVJ**  **N132A** | **1FQG**  **E166N** |
| --- | --- | --- | --- | --- | --- |
| 7-ACA derivative (3R,4S), ring A reactive^b^ | **Run 16:**  Hydrogen bonds: S70, K73, S130, E166, A237  Other: N170, S235, G236  Hydrophobic: Y105, V216, A237  Unfavorable: N132, N170 | **Run 86:**  Hydrogen bonds: S70, K73, S130, S235, A237, R244, N276  Other: Y105, S235  Hydrophobic: Y105, V216, A237 | **Run 32:**  Hydrogen bonds: K73, S130, N170, K234, S235, R244, V216  Other: S235  Hydrophobic: M272, A237  Unfavorable: N170  **Run 14:**  Hydrogen bonds: S70, K73, N170, A237, R275  Other: S235, A237  Hydrophobic: Y105, V216, A237 | **Run 53:**  Hydrogen bonds: S70, K73, A237, N276  Other: Y105, S235  Hydrophobic: Y105, A132, A237 | **Run 66:**  Hydrogen bonds: S70, S130, S235, A237, R243, N274  Other: S235  Hydrophobic: Y105, V216, A237  **Run 49:**  Hydrogen bonds: S130, N132, A237, R273, K274  Other: S235, G236  Hydrophobic: Y105, V216, A237 |
| 7-ACA derivative (3R,4S), ring B reactive^c^ | **Run 93:**  Hydrogen bonds: S70, K73, S130, N132, N170, K234, S235, A237, R244  Other: P167, S235, G236, E240,  Hydrophobic: Y105, P167, A237  **Run 63:**  Hydrogen bonds: S70, K73, N170, S235, A237, R244  Other: E104, S235, G236,  Hydrophobic: V216 | **Run 22:**  Hydrogen bonds: S70, K73, S130, N132, K234, S235, A237, R244  Other: S235, E240  Hydrophobic: Y105, P167, A237  Unfavorable: S130 | **Run 91:**  Hydrogen bonds: S70, K73, S130, N132, N170, A237, R244  Other: S235, E240  Hydrophobic: Y105, P167, A237  Unfavorable: S130 | **Run 24:**  Hydrogen bonds: S70, K73, S130, A237, R244  Other: K73, S130, E166, S235, G236, R244  Hydrophobic: A132, A237, M272  **Run 56:**  Hydrogen bonds: S70, K73  Other: G236, A237  Hydrophobic: Y105, A132 | **Run 59:**  Hydrogen bonds: S70, K73, S130, N132, N170, K234, A237, R243  Other: N132, S235, G236, E239  Hydrophobic: Y105, P167, A237 |

**Table S1 (cont.)**

| 7-ACA derivative (3S,4R), ring A reactive^b^ | **Run 47:**  Hydrogen bonds: S70, K73, A237, R275  Other: S130, K234, S235, G236  Hydrophobic: V216, A237 | **Run 7:**  Hydrogen bonds: S70, K73, K234, S235, A237, R244, N276  Other: S235  Hydrophobic: Y105, A237  Unfavorable: A237 | **Run 91:**  Hydrogen bonds: S70, K73, A237, R275  Other: S130, S235  Hydrophobic: V216, A237  Unfavorable: A237 | **Run 19:**  Hydrogen bonds: S70, A237  Other: G236  Hydrophobic: Y105, V216, A237  **Run 65:**  Hydrogen bonds: S70, S130, S235, A237, R244  Other: E166, S235  Hydrophobic: Y105, A132, A237, M272 | **Run 67:**  Hydrogen bonds: S70, A237, R243, N274  Other: S235  Hydrophobic: Y105, A237  **Run 49:**  Hydrogen bonds: S70, K73, A237, R273 (S235)  Other: K234  Hydrophobic: V216, A237, M270,  Unfavorable: A237 |
| --- | --- | --- | --- | --- | --- |
| 7-ACA derivative (3S,4R), ring B reactive^c^ | **Run 61:**  Hydrogen bonds: S70, K73, S130, N132, K234, S235, A237, R244  Other: S235, G236, M272  Hydrophobic: Y105, A237, G238 | **Run 77:**  Hydrogen bonds: S70, K73, S130, N132, K234, S235, A237, R244  Other: S235  Hydrophobic: A237, G238  Unfavorable: S130 | **Run 2:**  Hydrogen bonds: S70, K73, N132, K234, S235, A237  Other: S235, E240  Hydrophobic: Y105, P167, A237  **Run 39:**  Hydrogen bonds: S70, K73, S130, N132, S235, A237, R244  Other: S235, E240  Hydrophobic: Y105, A237  Unfavorable: S130 | **Run 54:**  Hydrogen bonds: S70, K73, N170, S235, A237, R244  Other: Y105, S235, A237  Hydrophobic: P167, V216, A237 | **Run 65:**  Hydrogen bonds: S70, S130, N132, K234, S235, A237, R243  Other: S235, G236, M270  Hydrophobic: Y105, A237 |

**Table S1 (cont.)**

| Ceftriaxone | **Run 26:**  Hydrogen bonds: S70, K73, S130, N132, P167, N170, K234, S235, A237, R244, N276  Other: E104, Y105, S235, G236, R244,  Hydrophobic: Y105, G238, M272  Unfavorable: V216, A237  **Run 60:**  Hydrogen bonds: S70, K73, S130, R244, A237  Other: Y105, M129, N170, S235, G236, R244  Hydrophobic: Y105, A237, M272  Unfavorable: N276 | **Run 21:**  Hydrogen bonds: S70, K73, S130, N132, N170, K234, S235, A237, E240, R244, N276,  Other: E104, S235, R244  Hydrophobic: Y105, P167  Unfavorable: A237 | **Run 46:**  Hydrogen bonds: S70, K73, S130, N132, K234, S235, A237, R244, N276  Other: E104, P167, R244,  Hydrophobic: Y105, P167  **Run 28:**  Hydrogen bonds: S70, K73, S130, N132, N170, K234, S235, A237, R244, N276  Other: E104, S235  Hydrophobic: Y105, A237, M272 | **Run 35:**  Hydrogen bonds: S70, K73, S130, K234, S235, A237, E240, R244, N276  Other: Y105, S235, M272,  Hydrophobic: P167, V216  **Run 45:**  Hydrogen bonds: K73, S130, N170, K234, A237, R244  Other: S130, G236, M272  Hydrophobic: Y105, A237  Unfavorable: A237 | **Run 94:**  Hydrogen bonds: S70, S130, N132, K234, A237, G239, R243, N274  Other: Y105, N132, S235  Hydrophobic: Y105, M270  Unfavorable: A237  **Run 4:**  Hydrogen bonds: S70, K73, S130, N132, N170, K234, A237, R243  Other: Y105, N132, P167, S235, G236  Hydrophobic: Y105, A237, M270  Unfavorable: A237 |
| --- | --- | --- | --- | --- | --- |

**Table S2: List of the interactions between 7-ACA derivative, ceftriaxone and the residues in the binding pocket of the class A β-lactamases different from TEM-1.** In this table are reported for each protein the interactions of the representative pose(s) reported in Table 3.

| **Ligand** | **3BLM**  **(PC-1 from *S.* *aureus,* WT)** | **3MKF**  **(SHV-1 from *K. pneumoniae,* WT)** | **4MBH**  **(SHV-1 from *K. pneumoniae,* E166A)** |
| --- | --- | --- | --- |
| 7-ACA derivative (3R,4S), ring A reactive^b^ | **Run 13:**  Hydrogen bonds: S70, K73, N132, Q237  Other: Y105, S130, S235, G236, R244,  Hydrophobic: I239  **Run 60**:  Hydrogen bonds: N132, Q237  Other: S235, G236  Hydrophobic: Y105 | **Run 20:**  Hydrogen bonds: S70, K73, T235, A237, R244, N276  Other: T235  Hydrophobic: Y105, M272  **Run 86:**  Hydrogen bonds: S70, K73, N132, G242, A237  Other: G236, E240  Hydrophobic: V216, A237 | **Run 17:**  Hydrogen bonds: S70, K73, S130, T235, A237, R244, N276  Other: T235, G236  Hydrophobic: A237, G238, E240 |
| 7-ACA derivative (3R,4S), ring B reactive^c^ | **Run 18:**  Hydrogen bonds: S70, K73, S130, N132, K234, Q237  Other: S235, Q237, I239  Hydrophobic: Y105  **Run 37:**  Hydrogen bonds: S70, K73, N214, S235, Q237  Other: S235  Hydrophobic: A104, I167, I239  Unfavorable: N132, S235 | **Run 41:**  Hydrogen bonds: S70, S130, N132, N170, K234, T235, A237, R244  Hydrophobic: A237 | **Run 58:**  Hydrogen bonds: S70, K73, S130, N132, A237  Other: T235, G236  Hydrophobic: Y105, A237 |

**Table S2 (cont.)**

| 7-ACA derivative (3S,4R), ring A reactive^b^ | **Run 100:**  Hydrogen bonds: S70, K73, Q237 Other: S235  Hydrophobic: I167, I239 | **Run 11:**  Hydrogen bonds: S70, K73, N132, A237, R275, Y105  Other: T235, G236  Hydrophobic: A237  Unfavorable: A237  **Run 69:**  Hydrogen bonds: S70, K73, N132, N170, A237  Other: T235, G236, R244  Hydrophobic: V216, A237  Unfavorable: A237 | **Run 13:**  Hydrogen bonds: S70, K73, N132, T235, A237, R244  Other: S130, T235, G236  Hydrophobic: V216, A237  **Run 71:**  Hydrogen bonds: S70, K73, N132, A237, R275  Other: T235, G236, R244  Hydrophobic: V216, A237 |
| --- | --- | --- | --- |
| 7-ACA derivative (3S,4R), ring B reactive^c^ | **Run 23:**  Hydrogen bonds: S70, K73, Y105, S130, K234, Q237, S235  Other: S235  Hydrophobic: Y105, I239  **Run 66:**  Hydrogen bonds: S70, K73, Y105, S130, K234, Q237, S235  Other: S235  Hydrophobic: A104, Y105  Unfavorable: K73 | **Run 54:**  Hydrogen bonds: S70, K73, S130, N132, K234, T235, A237, R244  Other: T235, G236, E240  Hydrophobic: A237  Unfavorable: S130, A237  **Run 56:**  Hydrogen bonds: S70, K73, A237  Other: S130, T235, G236, A237  Hydrophobic: A237, G238, V216 | **Run 29:**  Hydrogen bonds: S70, N132, T235, A237, R244  Other: D104, T235, A237  Hydrophobic: Y105, V216, A237  **Run 5:**  Hydrogen bonds: S70, K73, N132, T235, A237, R244  Other: D104, T235  Hydrophobic: V216  **Run 79:**  Hydrogen bonds: S70, K73, S130, N132, A237  Other: T235  Hydrophobic: Y205, A237, G238, |

**Table S2 (cont.)**

| Ceftriaxone | **Run 94:**  Hydrogen bonds: S70, K73, Q237, R244  Other: S216, S235  Hydrophobic: Y105  **Run 16:**  Hydrogen bonds: S70, K73, S130, N132, Q237, R244  Other: S216, S235, G236  Hydrophobic: I167 | **Run 62:**  Hydrogen bonds: S70, K73, S130, N132, T167, K234, T235, A237, R244, N170, E240  Other: M129, V216, T235, G236  Hydrophobic: V216, M129  **Run 82:**  Hydrogen bonds: S70, K73, S130, N132, T167, K234, T235, A237, R244, E240  Other: T235, G236, M272  Hydrophobic: A237 | **Run 55:**  Hydrogen bonds: S70, K73, A237, R244  Other: T235, G236  Hydrophobic: Y105, A237  **Run 38:**  Hydrogen bonds: S70, K73, K234, T235, A237, E240, R244  Other: T235, G236  Hydrophobic: Y105, A237 |
| --- | --- | --- | --- |

**Table S3: List of the interactions between 7-ACA derivative, ceftriaxone and the residues in the binding pocket of the class C and D β-lactamases.** In this table are reported for each protein the interactions of the representative pose(s) reported in Table 4.

| **Ligand** | **4KZ7**  **AmpC from *E. coli***  **WT** | **1L0F**  **AmpC from *E. coli***  **N152H** | **3LCE**  **OXA-10 from *P. aeruginosa***  **WT** | **4JF5**  **OXA-23 from *A. baumanii***  **WT** |
| --- | --- | --- | --- | --- |
| 7-ACA derivative (3R,4S), ring A reactive^b^ | **Run 84:**  Hydrogen bonds: K67, Q120, N152, G320  Other: A220  Hydrophobic: Y150, Y221, L293, A318 | **Run 79:**  Hydrogen bonds: K67, Y150, H152, R204, A318, G320, N343  Other: H152, K315, T316, G317, T319, A220  Hydrophobic: A318 | **Run 92:**  Hydrogen bonds: S67, S115, T206, F208, R250, K251  Other: T206, R250  Hydrophobic: A66, M99, L155, L247  **Run 52:**  Hydrogen bonds: S67, Q101, Q113, F208, R250  Other: Q113, T206  Hydrophobic: V117  Unfavorable: Q113 | **Run 95:**  Hydrogen bonds: S79, K82, K216, T217, W219, R259, N260  Other: S126  Hydrophobic: A256  **Run 82:**  Hydrogen bonds: S79, Q132, W219  Other: P129, G218, R259  Hydrophobic: A127, L166, M221  Unfavorable: W219 |
| 7-ACA derivative (3R,4S), ring B reactive^c^ | **Run 1:**  Hydrogen bonds: S64, Q120, N152, N289, K315, T316, A318, N343, N346  Other: A220  Hydrophobic: V211, A318 | **Run 72:**  Hydrogen bonds: S64, N289, A318, N346  Other: Y150, A220, G317 Hydrophobic: L293  **Run 57:**  Hydrogen bonds: S64, Q120, H152, N289, A318, N346  Other: H152, A220, G317  Hydrophobic: A318 | **Run 17:**  Hydrogen bonds: S67, S115, K205, T206, F208, R250  Other: W102, V117, T206  Hydrophobic: L155, F208  Unfavorable: S115 | **Run 36:**  Hydrogen bonds: S79, K82, L125, T217, R259  Other: K82, M221, D222,  Hydrophobic: L166, A220  **Run 40:**  Hydrogen bonds: K82, A127, W165, R259  Other: G218  Hydrophobic: A127, L166, A256 |

**Table S3 (cont.)**

| 7-ACA derivative (3S,4R), ring A reactive^b^ | **Run 95:**  Hydrogen bonds: S64, S212, Y221, A318, G320  Other: A220, T319  Hydrophobic: Y221, L293, A318 | **Run 93:**  Hydrogen bonds: S64, K67, H152, S212, A318, G320  Other: Q120, A220, K315, T316, T319  Hydrophobic: V211, Y221, T316, G317, A318 | **Run 12:**  Hydrogen bonds: S67, K*70, S115, T206, R250, K251  Other: T206, G207, R250  Hydrophobic: M99, W102, V117, L155, L247  **Run 48:**  Hydrogen bonds: S67, Q101, K205, T206, R250  Other: M99, T206, G207  Hydrophobic: A66, V114, V117, L155 | **Run 25:**  Hydrogen bonds: S79, K82, K216, T217, W219, R259  Other: R259  Hydrophobic: L166, M221, A256  **Run 95:**  Hydrogen bonds: A127, Q132, W219  Other: K216, G218  Hydrophobic: L166, T217  Unfavorable: A127 |
| --- | --- | --- | --- | --- |
| 7-ACA derivative (3S,4R), ring B reactive^c^ | **Run 51:**  Hydrogen bonds: S64, N152, N289, K315, T316, A318, N343, N346  Other: S212, A220  Hydrophobic: V211, A318, T319, G320  **Run 42:**  Hydrogen bonds: Q120, N152, A318, N343  Other: A220, G317  Hydrophobic: L219, Y221 | **Run 72:**  Hydrogen bonds: H152, N289, T316, A318, N346, R349  Other: H152, A220, T316, N343  Hydrophobic: A318  **Run 33:**  Hydrogen bonds: S64, G320  Other: A220, G317, T319,  Hydrophobic: Y221, H152, P345 | **Run 99:**  Hydrogen bonds: S67, S115, T206, F208, R250  Other: W102, T206, V211  Hydrophobic: M99, F208  Unfavorable: S115  **Run 8:**  Hydrogen bonds: S67, T206, F208, R250  Other: T206, M99, W102  Hydrophobic: A98, V117 | **Run 69:**  Hydrogen bonds: S79, K82, S126, W219, D222, R259  Other: G218, D222  Hydrophobic: L166, V167, M221 |

**Table S3 (cont.)**

| Ceftriaxone | **Run 10:**  Hydrogen bonds: S67, Q120, T316, A318, N343, N346  Other: A220, N289, G317  Hydrophobic: L119, Y221, A318  Unfavorable: T316, N343 | **Run 87:**  Hydrogen bonds: S64, K67, H152, E272, N289, K315, N343  Other: H152, A220, G317, N346  Hydrophobic: L119, Y150, H152, A292, L293 | **Run 1:**  Hydrogen bonds: S67, Q113, L155, K205, T206, F208, R250  Other: W102, T206  Hydrophobic: L155  **Run 44:**  Hydrogen bonds: S67, K205, T206, F208  Other: M99, V114, S115, L155, T206, F208  Hydrophobic: F208 | **Run 52:**  Hydrogen bonds: K82, L125, V128, Q132, W165  Other: W219, K216  Hydrophobic: A127, M221  **Run 35:**  Hydrogen bonds: K82, W165, K216, M221, D222  Other: T217, G218, A220  Hydrophobic: L166, M221, A256  Unfavorable: W219 |
| --- | --- | --- | --- | --- |

**Figure S1: Superposition between the representative run obtained by covalent self-docking (red) and the crystallographic structure of the complex (blue) of TEM-1 β-lactamase with penicillin (PDB file; 1FQG). The RMSD calculated on the two structures is 0.072 Å.**
